# Interrelationships Among Physical Fitness, General Motor Coordination, and Soccer-Specific Technical Skills in Youth Soccer Players

**DOI:** 10.3390/sports14060233

**Published:** 2026-06-05

**Authors:** Vanessa Rocco, Stefano Amatori, Roberto Bensi, Elvira Padua, Bruno Ruscello, Sergiu Vlad Lazau, Piero Tamagnini, Maria Chiara Ricciotti, Stélia Xavier, Marco Bruno Luigi Rocchi, Davide Sisti, Fabrizio Perroni

**Affiliations:** 1 Department of Theoretical and Applied Sciences, eCampus University, 22060 Novedrate, Italy; roccovanessa@yahoo.it; 2 Department of Biomolecular Sciences, University of Urbino Carlo Bo, 61029 Urbino, Italy; roberto.bensi@uniurb.it (R.B.); s.lazau@campus.uniurb.it (S.V.L.); p.tamagnini@campus.uniurb.it (P.T.); m.ricciotti1@campus.uniurb.it (M.C.R.); marco.rocchi@uniurb.it (M.B.L.R.); davide.sisti@uniurb.it (D.S.); fabrizio.perroni@uniurb.it (F.P.); 3 Department of Human Sciences and Promotion of Quality of Life, “San Raffaele” Open University of Rome, 00166 Rome, Italy; elvira.padua@uniroma5.it (E.P.); bruno.ruscello@uniroma5.it (B.R.); 4 Higher School of Sports Sciences, Eduardo Mondlane University, Maputo City 1102, Mozambique; steliax27@gmail.com

**Keywords:** coordinative abilities, soccer, performance, young athletes

## Abstract

Soccer performance is characterized by high motor and cognitive complexity, resulting from the interaction between, among others, physical and technical components. However, evidence regarding the relationships among physical performance, motor coordination and soccer-specific technical remains limited. Therefore, this cross-sectional study aimed to investigate the associations among these domains in youth soccer players. Forty-nine male U15 participants (age: 14.3 ± 0.5 years) underwent anthropometric assessments, physical fitness testing (10 m, 30 m sprint, CMJ, YYIRT1), a general motor coordination test (Harre Circuit Test), and soccer-specific technical evaluation (F-MARC test battery). Associations among variables were assessed using Spearman correlations and exploratory principal component analysis (PCA) based on a Spearman correlation matrix with oblimin rotation. Significant associations emerged between general motor coordination, physical performance variables, and several soccer-specific technical skills. The PCA identified three partially overlapping components, cumulatively explaining about 70% of the variance, highlighting the multidimensional and interconnected nature of soccer-related performance capacities. General motor coordination demonstrated relevant loadings in both coordinative/technical and physical-performance-oriented domains. These findings suggest that youth soccer performance should not be interpreted through isolated physical or technical characteristics, but rather as the result of interactions among coordinative, neuromuscular, and technical factors. Consequently, multidimensional and individualized training approaches integrating physical, coordinative, and technical stimuli may represent relevant strategies for youth soccer development.

## 1. Introduction

Soccer is a sport characterized by high motor and cognitive complexity, involving dynamic and intermittent movements such as kicking, sprinting, tackling, and jumping, which require strength, balance and neuromuscular coordination [1]. Accordingly, soccer-related performance can be considered a multidimensional construct involving, among others, physical, coordinative, and technical components [2]. Among physical qualities, lower-limb power has been associated with superior sprinting, jumping and change-of-direction performance [3]. However, physical fitness components (i.e., endurance, strength and speed) alone may not be sufficient for optimal player development. Coordinative abilities—intended as the capacity to organize, control, and adapt movement patterns [4]—also appear to play an important role in soccer performance [5,6]. Components such as spatial orientation, movement differentiation, reaction ability, motor adaptation, and motor coupling are particularly relevant in soccer, because they underpin the ability to rapidly adjust movements in response to changing environmental and task constraints [4,7]. These abilities are likely involved in multidirectional actions such as changes in direction, ball control, and the execution of technical skills under time pressure and opponent interaction, while potentially enhancing the application of strength and power in soccer-specific situations [8]. Technical skills—also referred to as soccer-specific motor skills (e.g., passing, shooting, heading, controlling, and dribbling)—are frequently operationalized through measures of accuracy, precision and ball velocity [9]. From this perspective, physical fitness components may provide the quantitative basis for performance, whereas coordinative abilities may contribute to movement quality, adaptability, and execution efficiency [10]. Overall, soccer-related performance results from continuous interaction among physical, coordinative, technical, and contextual factors during gameplay.

Despite increasing interest in youth soccer development, the existing literature still lacks sufficient evidence regarding the interaction between physical fitness components, general motor coordination, and technical skills in young players. From both theoretical and practical perspectives, coordinative abilities may represent fundamental prerequisites for the effective execution and adaptation of movement patterns [4], potentially influencing not only technical skills, but also the expression of physical capacities through more efficient force application and movement organization during tasks such as sprinting, jumping, and change-of-direction actions. However, in practical settings within youth sports academies, training approaches often appear to emphasize performance outcomes more than the comprehensive development of fundamental coordinative foundations. Moreover, limited evidence is available regarding how these relationships may be influenced by biological maturation during this developmental stage. A clearer understanding of the relationships among coordinative abilities, physical fitness components, and technical skills could therefore contribute to talent identification processes and support the prescription of age- and maturation-appropriate training interventions.

To assess general motor coordination, we selected the Harre circuit test (HCT), originally developed by German sports scientist Dietrich Harre to evaluate whole body coordination [11]. The HCT that has been previously defined as a suitable tool to evaluate youth players’ ability to coordinate complex dynamic movements [6,12]. Specifically, the test evaluates dynamic motor coordination, coordinative abilities and cognitive capabilities, as it requires the rapid and efficient execution of multidirectional motor tasks involving actions such as somersaulting, hopping, running, and turning, integrating several joints and varying force demands [6,13,14].

Therefore, the aim of this cross-sectional study was to analyze the relationship between physical fitness components, coordinative abilities, and technical skills in youth soccer players, while considering the potential influence of maturation status. We hypothesized that superior coordinative abilities (i.e., lower execution times in the HCT) would be associated with better physical performance variables and soccer-specific technical skills, reflecting the potential role of general motor coordination as a foundational component underlying soccer-related performance capacities.

## 2. Materials and Methods

### 2.1. Participants

A total of 49 young male soccer players competing in the under-15 category from the same provincial-level Italian club were examined. Of these, 6 were goalkeepers, 16 were defenders, 15 were midfielders, and 12 were forwards. Inclusion criteria were: (i) active participation in official training sessions and matches, (ii) absence of musculoskeletal injury in the previous 3 months, and (iii) regular training attendance (>80%). All participants competed in a non-elite provincial league and belonged to the same chronological age category (U15). During the data collection period, participants trained three times per week (approximately for 1.5 h per session) and played one official match on the weekend (two halves of 35 min). No structured resistance training program was systematically performed. Prior to testing, participants completed a 2-week familiarization period to minimize potential learning effects associated with the testing procedures. All training sessions during this period followed a standardized structure consisting of approximately 10 min of joint-mobility exercises, 10 min of dynamic warm-up activities, and 10 min of ball-based activation, followed by the main training content (technical, tactical, and small-sided game activities). Before the start of the study, all participants and their legal guardians were contacted and informed about the study procedures. Written informed consent was obtained from all players and their parents or guardians before the start of the study. The Ethics Committee of eCampus University (no. 17/2025) approved this study.

### 2.2. Procedures

This cross-sectional observational study was conducted during the competitive season, during March and April 2026. All testing session were conducted under standardized conditions at the same time of day (between 4:00 and 6:00 PM). Participants were instructed to maintain their usual dietary habits and to avoid strenuous physical activity in the 24 h preceding testing. All players were free from injury at the time of assessment and fully participated in regular training and competition during the testing period. However, external training load, match exposure, and accumulated fatigue were not objectively monitored prior to data collection. Participants were asked to wear the same type of clothing and footwear (soccer boots, except for the CMJ test), and were verbally encouraged to give a maximal effort during all testing sessions. Anthropometric measurements (stature, body mass) were taken with an electronic scale and a stadiometer (Seca 702, Seca GmbH and Co. KG, Hamburg, Germany) with a precision of ±0.1 kg and ±0.1 cm, respectively. The maturity status of players was estimated using the equation proposed by Moore et al. [15], which estimates age at peak height velocity (PHV) and maturity offset (MO). Players were classified as pre-PHV (MO < −0.5 years before PHV), circa-PHV (MO between −0.5 and 0.5 years from PHV) and post-PHV (MO > 0.5 years after PHV). The physical fitness assessment included the 10 m and 30 m linear sprint tests, countermovement jump (CMJ), and the Yo-Yo Intermittent Recovery Test Level 1 (YYIRT1). Soccer-specific technical skills were assessed using the FIFA F-MARC test battery, which comprised eight different skill tests [16]. Finally, general motor coordination was evaluated using the HCT [5,6,17]. All tests were performed on four separate occasions, with at least 48 h between sessions. The order of testing was kept consistent across participants to reduce interindividual variability: day 1, CMJ, 10 m and 30 m sprints; day 2, HCT; day 3, F-MARC battery; day 4, YYIRT1. All testing sessions were carried out on a regular (100 × 60 m) artificial grass soccer pitch. Before the evaluations, players underwent a 15 min warm-up consisting of low-intensity running, dynamic stretching, skipping drills, progressive accelerations, and jumping exercises of increasing intensity before the physical tests. Unless otherwise specified, participants completed three trials for each test, with a 3 min passive recovery period between trials.

### 2.3. Measurements

#### 2.3.1. Sprint Performance

Participants completed separate linear sprints over distances of 10 and 30 m using photocells (Witty, Microgate, Trieste, Italy). For each distance, participants completed three maximal sprint trials separated by 3 min of passive recovery, and the best performance was retained for analysis. Participants started from a standing static position, approximately 0.5 m behind the first timing gate to avoid early triggering of the photocells. Sprint time was recorded between the starting gate and the corresponding finishing gate positioned at either 10 m or 30 m from the start line. Regarding the reliability of these tests, a systematic review reported intraclass correlation coefficients (ICC) > 0.75, for both intraday and inter-day reliability in sprint assessments performed in team-sport athletes [18].

#### 2.3.2. Endurance Capacity

Endurance capacity was assessed using the YYIRT1 according to the procedures suggested by Krustrup et al. [19]. The test consists of repeated 2 × 20 m runs back and forth between the starting, turning, and finishing lines at a progressively increasing speed controlled by audio beeps from a tape recorder. Players ran until they could not keep pace with the audio beeps. After each running bout, participants were allowed a 10 s active rest interval to recover, during which they jogged around a cone placed 5 m behind the starting line. The test ended when participants either failed twice to reach the line in time with the audio signal or voluntarily stopped because of exhaustion despite verbal encouragement. Total covered distance was measured in meters. All participants were familiarized with the testing procedures before formal testing. Regarding reliability, Fanchini et al. [20] reported good reliability for the YYIRT1, with an ICC of 0.78 (95% CI: 0.61–0.89).

#### 2.3.3. Lower-Extremity Explosive Strength

Lower-extremity explosive strength was assessed using the countermovement jump (CMJ). Participants performed a maximal vertical jump without arm swing on a hard, flat surface. Participants were instructed to keep their hands on their hips from the starting position through the countermovement phase, jump, and until landing. Jump height (in cm) was measured using an optical detection system (Optojump, Microgate, Bolzano, Italy). Each participant performed three trials separated by 2 min of passive recovery, and the best performance was retained for analysis. Regarding reliability, Slinde et al. [21] reported high test–retest reliability for CMJ performance, with ICC values ranging from 0.80 to 0.98.

#### 2.3.4. General Motor Coordination

**Figure 1:**
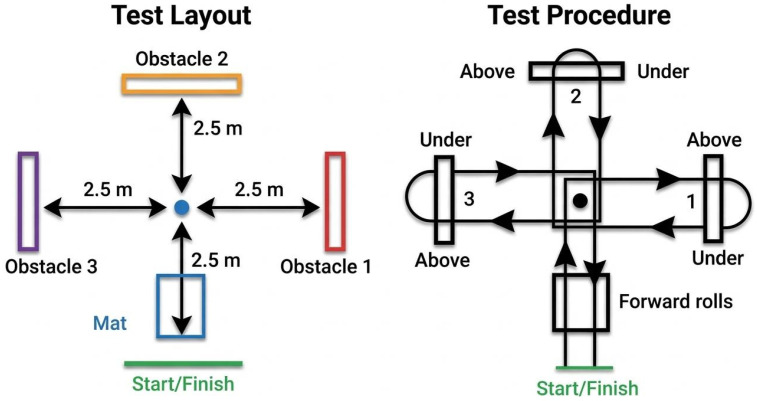
Harre circuit test.

General motor coordination is defined as the capacity to execute different controlled movements, irrespective of sport-specific skills [5,6], and in this study was assessed using the Harre circuit test (HCT). The HCT was selected because it requires rapid execution of multidirectional whole-body movements involving spatial orientation, movement adaptation, agility, and dynamic coordination [17]. Although the HCT also includes relevant physical demands related to agility and rapid movement execution, it has previously been proposed as a useful assessment of integrated coordinative performance in youth athletes [22]. The test started with the execution of a forward roll (performed once after the start), followed by three consecutive passages over and under three obstacles (Figure 1). Execution time was recorded using photocells (Witty, Microgate, Bolzano, Italy), with timing gates positioned on the starting line at a height of approximately 1 m. Any execution errors during the circuit were noted (e.g., contact with the obstacles or the cone positioned at the center of the course). Regarding reliability, excellent ICC values (ICC > 0.955) were previously observed in the HCT [6].

#### 2.3.5. Soccer-Specific Technical Skills

The F-MARC (FIFA Medical Assessment and Research Center) test battery was used to obtain information on soccer-specific technical skills through a standardized set of field-based tasks [16,23]. The battery included: (a) right- and left- foot juggling, (b) body juggling, (c) speed dribbling, (d) long passing, (e) short passing, (f) shooting a stationary ball, (g) shooting from a pass, and (h) heading from different positions. Juggling tests evaluate ball control under continuous touch conditions, using both feet and body. Speed dribbling performance was assessed through a timed slalom course requiring rapid changes in direction while maintaining ball control. Passing tests evaluated accuracy and precision over short and long distances toward predefined targets. Shooting tasks assess both accuracy and ball velocity under stationary and dynamic conditions, whereas heading tests evaluate control and directional accuracy from different ball-delivery positions. Depending on the specific task, performance outcomes were expressed either as execution time (e.g., dribbling speed) and/or scores. A detailed description of test execution, number of trials for each test, and scoring is reported in Rosch et al. [16]. A 3 min recovery interval separated trials, and a 5 min rest period separated different tests. For bilateral tasks (right- and left-foot juggling and shooting tests), the best score obtained between limbs was retained for analysis to represent maximal technical performance capacity under optimal execution conditions. Body juggling performance was calculated as the mean of the three trials, as this task showed low trial-to-trial variability and was intended to capture consistent whole-body control performance rather than maximal output. Given the heterogeneous nature of the F-MARC test components and their differing scoring scales, all variables were standardized (z-scores) prior to analyses to ensure comparability and to prevent scale-dependent weighting effects. Regarding reliability, Padrón-Cabo et al. [24] reported high reliability for most F-MARC tests, with ICC generally exceeding 0.80.

### 2.4. Statistical Analysis

Data are presented as mean ± standard deviation or median [first-third quartile], as appropriate. Normality of the variables was assessed using the Shapiro–Wilk test. Because several variables, particularly some outcomes derived from the F-MARC battery, were ordinal or discrete in nature, and because most variables were not normally distributed, nonparametric statistical procedures were preferred. Prior to analysis, the dataset was inspected for missing values. Approximately 3% of observations were missing due to random absences of players during testing sessions. Missing data were handled using k-nearest neighbors imputation with the *VIM* package [25], after variable standardization. An initial value of k = 5 nearest neighbors was selected as a compromise between local sensitivity and stability in imputation procedures. Sensitivity analyses using alternative values (k = 3 and k = 7) yielded highly comparable imputed datasets and analytical results. Given the low proportion of missing data, the choice of k was expected to have minimal influence on the imputed data. Differences in physical performance, general motor coordination and soccer-specific skills tests between maturation groups were exploratorily compared using the Kruskal–Wallis test, with Benjamini–Hochberg correction applied to control for multiple comparisons. When significant main effects were detected, pairwise post hoc comparisons were performed using Dunn’s test with Bonferroni correction. Effect sizes were calculated as eta squared based on the Kruskal–Wallis H statistic (*η^2^_H_*) according to the formula proposed by Tomczak and Tomczak [26] and interpreted as small (*η*^2^ < 0.06), medium (0.06 ≤ *η*^2^ < 0.14), and large (*η*^2^ ≥ 0.14) [27]. Time-based measures (10 m and 30 m sprint, HCT and F-MARC speed dribbling), for which lower values represent better performance, were reversed to ensure consistent interpretability across performance indicators. Associations among physical performance (10 m and 30 m sprint, YYIRT1, CMJ), coordination-oriented motor performance (HCT), and soccer-specific skills (F-MARC test battery) variables were examined using Spearman’s rank correlation coefficients, and interpreted as follows: *r_s_* < 0.10, negligible; 0.10 ≤ *r_s_* < 0.39, weak; 0.40 ≤ *r_s_* < 0.69, moderate; 0.70 ≤ *r_s_* < 0.89, strong; *r_s_* ≥ 0.90 = very strong [28]. *p*-values associated with correlation analyses were adjusted using the Benjamini–Hochberg correction procedure, and confidence intervals were additionally calculated for all correlation coefficients. To explore the underlying structure of the performance variables and reduce dimensionality, an exploratory principal component analysis (PCA) was performed on standardized variables using a Spearman correlation matrix. The suitability of the data for PCA was assessed using the Kaiser–Meyer–Olkin measure of sampling adequacy and Bartlett’s test of sphericity. The number of retained components was determined by considering the eigenvalue structure, the scree plot, the proportion of variance explained, and the interpretability of the resulting loading pattern. Given the conceptual and statistical interrelationships among physical, technical, and coordinative domains, component loadings were rotated using the oblique oblimin rotation method. Variables with absolute loadings ≥ 0.40 were considered to meaningfully contribute to a component. Given the relatively small sample size in relation to the number of variables, the PCA was not intended to confirm latent constructs, but rather to identify empirical clustering patterns among the measured variables and should be considered exploratory in nature. Therefore, component labels were assigned descriptively according to the variables with the highest loadings on each component. To further assess the stability of the PCA solution, a bootstrap resampling procedure was conducted using 1000 bootstrap samples generated with replacement. In addition, a sensitivity analysis excluding goalkeepers was performed to evaluate the potential influence of position-specific characteristics on the correlation structure and PCA solution. All statistical analyses were performed in RStudio (version 2025.05.0+496, Posit Studio, PBC, Boston, MA, USA). Statistical significance was set at α = 0.05.

## 3. Results

A total of 49 players (age: 14.3 ± 0.5 years; body mass: 57.4 ± 15.0 kg; height: 165.7 ± 9.5 cm; BMI: 20.7 ± 4.1 kg/m^2^) completed the proposed test batteries. One player was excluded from the initial sample because he was absent for more than one testing session. The MO was 0.41 ± 1.40 years, with an age at PHV estimated at 13.92 ± 1.37 years; accordingly, players were classified as pre-PHV (n = 3), circa-PHV (n = 19) and post-PHV (n = 27). The results obtained for the different tests among the maturation groups are presented in Table 1. No significant differences between maturation groups were found, except for the frontal heading test (*p* = 0.0162, *η*^2^ = 0.25), in which test post-PHV players showed significantly better performances compared to pre-PHV (*p* = 0.027) and circa-PHV (*p* = 0.007). However, given the very small number of participants in the pre-PHV group, the absence of statistically significant maturation-related differences for most variables should be interpreted cautiously, as the analyses may have been underpowered and susceptible to type II error.

**Figure 2:**
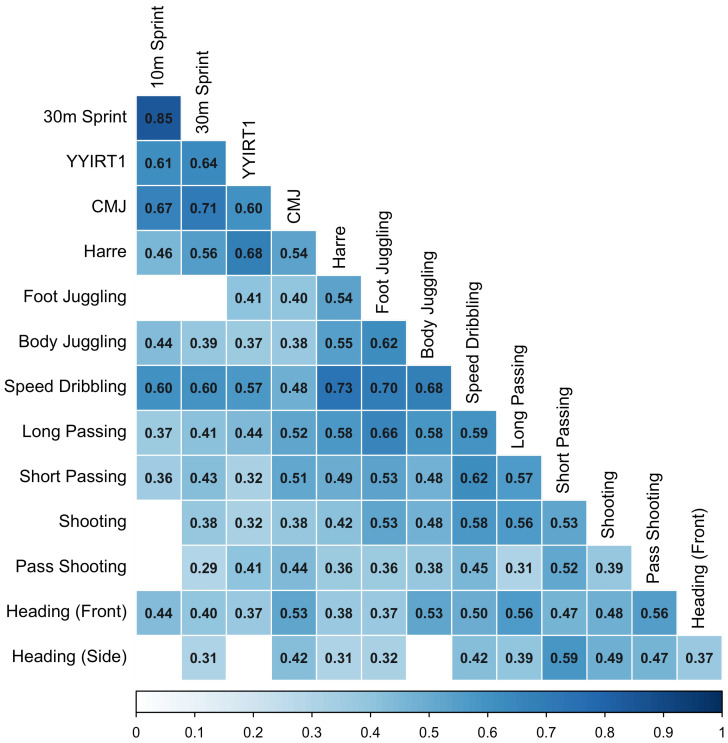
Spearman correlation matrix among performance variables (n = 49). Colored cells represent correlation strength and direction, while numerical values indicate correlation coefficients. Non-significant correlations (*p* > 0.05) are reported in white cells.

Figure 2 presents a correlation matrix of the results of all the tests performed. For interpretative consistency, time-based variables (10 m sprint, 30 m sprint, HCT, and speed dribbling) were reversed prior to the correlation analyses; therefore, higher values corresponded to better performance for all variables included in the correlation matrix. Overall, moderate to strong correlations (0.4 < *r_s_* < 0.8) were observed between almost all F-MARC tests, with some exceptions particularly between foot juggling, shooting from a pass and heading, for whose weak correlations were detected. Similarly, better HCT performance was significantly associated with better performance in the F-MARC tests, in particular with the speed dribbling test (*r_s_* = 0.73) suggesting that general coordination and technical skills are interconnected. Among the physical components, as expected the results in the 10 m and 30 m sprints, YYIRT1 and CMJ were strongly correlated among each other (*r_s_* > 0.6), and with HCT (0.46 < *r_s_* < 0.68). The complete correlation matrix reporting Spearman’s rho coefficients, confidence intervals, and adjusted *p*-values has been included as Supplementary Material (Table S1).

To explore the underlying structure of the performance variables and reduce dimensionality, a principal component analysis was applied. The Kaiser–Meyer–Olkin measure indicated adequate sampling adequacy (KMO = 0.85), and Bartlett’s test of sphericity was significant (*p* < 0.001), indicating that sufficient correlations existed among variables to proceed with PCA. The analysis identified three components with eigenvalues > 1. Inspection of the scree plot and the cumulative explained variance supported the retention of three components, which together explained 70.2% of the total variance. To facilitate interpretation, component loadings were rotated using the oblique oblimin rotation method. Only loadings with an absolute value ≥ 0.40 were considered meaningful and are reported in Table 2. The complete loadings are reported as Supplementary Material (Table S2). The first component (27% of variance) was primarily characterized by high loadings for juggling and speed-dribbling, together with long passing and shooting variables, suggesting a domain related to coordination and ball-control abilities. The second component (24% of variance) showed high loadings for 10 m and 30 m sprint performance, YYIRT1 and CMJ, reflecting a domain associated with physical performance capacities. The third component (19% of variance) was mainly characterized by soccer-specific technical tasks requiring accuracy and precision, including short passing, pass shooting and heading performance, then suggesting a technical-skills domain. Interestingly, the HCT demonstrated relevant loadings on both the coordination-oriented and physical-performance components, suggesting that successful execution of this test may depend on both whole-body coordinative control and physical performance capacities. Similarly, also the shooting performance entered both in the first and third component. However, because the PCA was exploratory in nature, these components should be interpreted as statistical clustering patterns among partially overlapping performance domains rather than as definitive latent constructs.

Lastly, an additional analysis excluding goalkeepers (n = 6) yielded highly comparable correlation patterns and PCA structure, with the same three-component solution and similar loading distributions, suggesting that goalkeeper inclusion did not materially influence the results.

## 4. Discussion

Performance in contemporary soccer-specific contexts, is increasingly conceptualized as the outcome of a complex, integrated system, in which technical skills, biomechanical efficiency, postural stability, and physiological conditioning interact to influence match outcome. The present study aimed to investigate the relationships among physical performance components—including sprint, explosive strength and endurance—general coordination, and soccer-specific technical skills. The main findings revealed that general motor coordination (i.e., HCT performance) was significantly associated with both physical performances and some soccer-specific skills, such as speed dribbling and juggling, suggesting that whole-body coordination may represent an important factor associated with soccer-specific technical performances. Moreover, the PCA emphasized this relationship, with the HCT showing relevant loading both in the component related to ball-control abilities (PC1) and in the component associated with the physical performances (PC2). However, the cross-sectional design does not allow causal inferences; therefore, general motor coordination should not be interpreted as a direct determinant of technical performance. It is equally plausible that the observed association reflects unmeasured factors related to sport participation and skill development. For example, greater soccer exposure, practice volume, or task-specific training may contribute to the concurrent development of both technical proficiency and coordinative abilities [5,29]. However, these variables were not assessed in the present study and therefore this interpretation remains speculative. Thus, the relationship between general motor coordination and technical performance is likely bidirectional and dynamically shaped by both motor development and other factors.

The associations found between general motor coordination (i.e., HCT) and sport-specific skills (i.e., F-MARC tests) are partially supported by previous evidence. Kokštejn and Musalek [30] observed moderate correlations (*r* = 0.62) between fundamental motor skills (evaluated using the Test for Gross Motor Development-2) and game specific motor skills (dribbling and shooting) in elite youth soccer players. Similarly, Kamandulis et al. [5] reported significant correlations (*r* ranging from 0.52 to 0.76) between a general coordination test (20 m run with obstacles) and the basketball-specific coordination (Illinois agility run with dribbling) in a sample of youth basketball players. Notably, authors reported that correlations were weaker in the older age groups (i.e., 16–17 years old), suggesting that the importance of general coordination decreases after sport-specific skills are mastered. Likewise, Chagas et al. [31] reported that gross motor coordination explained approximately 23% of the variance in volleyball-specific technical performance among adolescent non-athletes, suggesting that coordinative abilities may contribute substantially to sport-specific motor execution even outside highly specialized athletic populations.

Several theoretical frameworks support these findings by proposing that gross motor coordination and fundamental movement skills represent the “building blocks” for the acquisition of more complex sport-specific skills [32]. From this perspective, coordinative abilities may facilitate the organization, timing, and adaptation of movement patterns required for efficient technical execution. Ball-control tasks such as dribbling and juggling require continuous integration of postural regulation, spatial orientation, movement differentiation, and rapid sensorimotor adjustments in response to changing environmental constraints. Therefore, players with better general coordination may exhibit superior movement efficiency and adaptability during technically demanding tasks. Only a few studies have focused on the analysis of sport-specific technical skills in young soccer players [23]. In our study, moderate correlations between juggling technique and shot accuracy were found. Higher juggling performance may reflect superior neuromuscular coordination of the foot, ankle, and leg, thereby possibly improving shooting accuracy, proprioceptive sensitivity and balance control. Previous studies [33] have highlighted that shooting velocity and accuracy are associated with overall body stability and trunk angle during single-leg support (fundamental elements in juggling). Similarly, effective balance control and dynamic postural adaptation may contribute to successful heading performance. Evidence on soccer heading performance [34] suggests that efficient body positioning and stable support patterns contribute to improved ball trajectory control and execution quality. Moreover, previous studies [35] on bilateral transfer indicated that training one limb may positively influence the contralateral limb, supporting the idea that coordinative training may induce generalized neuromuscular adaptations extending beyond isolated technical actions. Training activities involving juggling and foot control may therefore contribute not only to sport-specific abilities, but also to the development of coordinative capacities such as rhythmic ability, kinesthetic differentiation, dynamic balance and motor creativity. Collectively, these capacities may contribute to performance similarities across several soccer-specific tasks, including rapid dribbling, shooting, passing techniques, and heading [23].

The observed associations between HCT performance and physical fitness variables may further support the hypothesis that coordinative abilities contribute not only to technical proficiency, but also to the efficient expression of physical capacities. In the PCA, PC2 included all physical performance tests (10 m sprint, 30 m sprint, CMJ and YYIRT1), along with the HCT. Although technical variables were not substantially represented within this component, the inclusion of the HCT suggests partial overlap between coordinative and physical performance domains. The HCT requires a complex sequence of dynamic movements, such as jumps, changes in direction, and rolls, all of which require rapid movement, dynamic coordination, balance, and advanced motor control [6]. Consequently, players with superior sprinting and jumping capacities may also exhibit more efficient execution of the HCT, because of enhanced neuromuscular performance and reduced ground contact times. This interpretation is partially supported by previous evidence. Pion et al. [36] and Rommers et al. [37] highlighted that motor coordination represents an important underlying component of agility and change-of-direction performance in team-sport athletes. Likewise, Menezes et al. [38] reported that motor coordination significantly influenced movement-response time, agility, and change-of-direction speed independently of maturity offset, suggesting that the transfer of physical capacities such as speed and power into efficient sport-specific movement execution may depend substantially on coordinative ability. According to these authors, coordination primarily influences the motor-execution component of agility rather than perceptual-decision processes, likely through more effective synchronization of sensory, neural, and muscular systems during movement regulation. The present findings are also partially consistent with longitudinal evidence reported in youth soccer players. Deprez et al. [39] found that non-sport-specific fundamental movement skills predicted explosive power development from childhood into young adulthood, while another paper from the same research group [40] reported that general motor competence was associated with subsequent soccer-specific aerobic performance in pubertal elite soccer players. These authors suggested that players with better coordinative foundations acquired during childhood may later benefit from more efficient movement organization and force transmission during adolescence.

Interestingly, a significant relationship also emerged between speed dribbling and the YYIRT1 performance. This association may be explained by several factors. One possible explanation is that unmeasured factors related to soccer participation, such as training history, accumulated practice volume, or playing experience, may contribute to both aerobic fitness and technical proficiency [23]. However, because these variables were not collected, this interpretation should be considered speculative. Furthermore, the YYIRT1 is a shuttle-based test that requires pacing strategies, repeated accelerations, decelerations, and the capacity to sustain high-intensity efforts under fatigue. Technical skills in soccer are frequently executed under fatigued conditions during matches; therefore, recovery capacity and the ability to maintain motor control under physiological stress may contribute to superior technical performance. Previous evidence supports this interpretation, indicating that endurance capacity may influence technical skill execution during repeated high-intensity efforts in youth soccer players [2,41].

Despite these associations, the present findings should not be interpreted as evidence that coordinative abilities represent isolated or dominant determinants of soccer performance. Several variables demonstrated relevant cross-loading patterns within the PCA, emphasizing the substantial overlap among physical, coordinative, and technical domains. For instance, speed dribbling likely depends not only on ball-control ability, but also on sprinting speed, agility, lower-limb power, and movement efficiency. Similarly, the HCT itself should not be considered a “pure” coordination assessment, as successful execution also depends on agility, speed–strength integration, and rapid movement execution. Therefore, the extracted PCA components should be interpreted as partially overlapping statistical clustering patterns rather than discrete latent constructs of soccer performance.

Notably, no significant differences in physical, coordinative, or soccer-specific technical outcomes between maturation groups were observed. However, these findings should be interpreted cautiously because the very small number of pre-PHV players substantially reduced statistical power. Previous evidence has shown that sprinting speed, agility, and strength are strongly influenced by biological maturation, with earlier maturing athletes generally outperforming later maturing peers. Conversely, some evidence suggests that coordinative abilities may be comparatively less sensitive to maturation-related changes and therefore may represent a more stable indicator of long-term athletic potential during adolescence. For example, Coelho e Silva et al. [29] reported that basketball technical skills appeared relatively independent of pubertal status, whereas Menezes et al. [38] found that the relationships between motor coordination and agility remained significant even after controlling for maturity offset. However, findings in this area remain inconsistent. In contrast with previous youth soccer studies by Figueiredo et al. [42] and Vandendriessche et al. [43], Rommers et al. [37] reported significant maturation-related effects on generic motor coordination performance. Therefore, coordinative development during adolescence should likely be considered a multifactorial and non-linear process influenced by the interaction of biological maturation, motor experience, and environmental constraints. Although training exposure may also play a role, this variable was not assessed in the present study. This aligns with evidence suggesting that coordinative abilities may show limited sensitivity to growth-related changes during adolescence and are more strongly influenced by environmental and task-specific factors than by maturation alone [10,30].

### Limitations

This study presents several limitations that should be considered when interpreting the findings. First, the exploratory cross-sectional design does not allow causal inferences to be drawn from the observed associations and limits the possibility of identifying underlying mechanisms. Accordingly, the findings should be interpreted as associative rather than causal, within the multifactorial context of youth soccer performance. In addition, the relatively small sample size reduces statistical power and limits the generalizability of the results. Although maturation status was considered, the very small pre-PHV subgroup represents an important limitation, reducing the robustness and interpretability of maturation-related comparisons. In addition, maturity status was estimated using a maturity offset equation [15], and the limitations associated with such predictive equations should be acknowledged. Previous literature has shown that these equations may present reduced accuracy at the individual level and may introduce classification errors, particularly in individuals close to PHV [44]. Consequently, some degree of misclassification cannot be excluded, especially considering the relatively small and uneven maturation groups observed in the present sample. Furthermore, the single-club design limits the external validity of the findings, and future studies should include larger multi-club samples across different competitive levels and with more balanced maturation distributions. Additional methodological considerations should also be acknowledged. Although positional role may influence performance characteristics, its impact in the present sample was likely limited because structured positional specialization had only recently been introduced, whereas earlier developmental stages were characterized by more generalized participation across playing positions. In addition, tactical and cognitive dimensions of soccer performance were not assessed and should be considered in future investigations to provide a more comprehensive understanding of youth soccer performance. The HCT should also be interpreted cautiously as a proxy measure of coordination because of its multifactorial nature. Performance in this test likely depends not only on coordinative abilities, but also on speed, agility, rapid changes in direction, and speed–strength integration and therefore may not fully isolate coordinative performance. Limitations specifically related to the PCA should also be acknowledged. Although the Kaiser–Meyer–Olkin statistic indicated acceptable sampling adequacy, the PCA was conducted on 14 variables in a sample of 49 participants, resulting in a relatively modest participant-to-variable ratio. Consequently, the stability and reproducibility of the extracted components may be limited. Moreover, the three retained components explained relatively similar proportions of variance, suggesting a distributed or partially overlapping component structure rather than a clearly dominant dimensional organization. Therefore, the identified components should not be interpreted as definitive latent constructs, but rather as exploratory statistical clustering patterns observed within the present dataset. To improve transparency, the scree plot was added as Supplementary Material and the PCA was explicitly framed as exploratory and hypothesis-generating. In addition, an exploratory bootstrap resampling procedure was performed to provide preliminary information regarding loading stability. Nevertheless, replication in larger and more balanced samples, ideally including validation procedures and independent cohorts, is necessary before stronger conclusions regarding the component structure can be drawn.

## 5. Conclusions

The present findings support the view that youth soccer performance develops in a multidimensional manner, through the interaction of multiple and partially overlapping domains, rather than through isolated physical or technical capacities. In particular, general motor coordination was associated with both physical performance variables and several soccer-specific technical skills, suggesting that coordinative abilities may contribute to the efficient execution of complex sport-specific actions during adolescence. The PCA further emphasized the multidimensional nature of soccer-related performance, identifying partially overlapping components integrating physical, coordinative, and technical characteristics rather than clearly separated domains. These findings reinforce the concept that technical proficiency in youth soccer should not be interpreted independently from the broader neuromuscular and coordinative context in which it develops. At the same time, because of the exploratory cross-sectional design, the observed relationships should be interpreted as associative rather than causal. It remains possible that unmeasured factors related to sport participation, including sport-specific exposure and practice volume, contribute to the concurrent development of technical proficiency, physical fitness, and coordinative abilities. However, because these variables were not directly assessed, this interpretation should be considered hypothetical. From a practical perspective, the results support the potential value of multidimensional and individualized training approaches during youth development. In particular, coordinative training and the development of general motor competence may represent relevant complementary targets alongside traditional physical and technical training strategies. Accordingly, training programs in youth soccer may benefit from integrating technical, coordinative, and physical stimuli rather than addressing these domains as independent components of performance. Nevertheless, the exploratory nature of the PCA, the relatively small sample size, and the uneven maturation-group distribution require cautious interpretation of the findings. Future longitudinal and intervention-based studies involving larger and more balanced samples are needed to clarify the causal relationships among coordinative abilities, physical fitness, biological maturation, and soccer-specific technical performance throughout youth development.

## Figures and Tables

**Table 1:** Descriptive data for physical performance, general motor coordination and soccer-specific skills tests are reported as median [Q1–Q3]; *p*-values and effect sizes are also reported.

	Pre-PHV (n = 3)	Circa-PHV (n = 19)	Post-PHV (n = 27)	*p*	*η^2^_H_* [95% CI]
**Physical performance**					
Sprint 10 m (s)	2.02 [2.00–2.05]	1.90 [1.90–2.10]	1.90 [1.80–2.00]	0.336	0.04 [−0.03–0.26]
Sprint 30 m (s)	5.03 [4.90–5.12]	4.80 [4.60–5.20]	4.70 [4.50–5.10]	0.592	−0.01 [−0.04–0.17]
YYIRT1 (m)	840.0 [640.0–1000.0]	760.0 [460.0–1080.0]	800.0 [420.0–1340.0]	0.963	−0.04 [−0.04–0.08]
CMJ (cm)	29.00 [25.80–29.20]	26.80 [22.00–30.90]	32.80 [26.80–35.50]	0.253	0.08 [−0.03–0.31]
**Motor coordination**					
Harre circuit test (s)	13.40 [12.60–13.80]	13.30 [12.50–15.10]	13.50 [12.30–14.80]	0.963	−0.04 [−0.04–0.10]
**Soccer-specific skills**					
Foot Juggling (n)	25.0 [25.0–25.0]	18.8 [8.0–25.0]	25.0 [8.5–25.0]	0.411	0.02 [−0.02–0.17]
Body Juggling (score)	1.0 [1.0–1.0]	1.0 [1.0–2.0]	1.0 [1.0–2.0]	0.663	−0.02 [−0.04–0.14]
Speed Dribbling (s)	22.0 [21.4–22.6]	22.5 [21.3–23.4]	21.6 [19.1–23.6]	0.723	−0.02 [−0.04–0.16]
Long Passing (score)	1.0 [1.0–1.5]	1.0 [0.0–2.0]	2.0 [1.0–3.5]	0.411	0.02 [−0.04–0.24]
Short Passing (score)	4.0 [3.5–5.5]	6.0 [4.0–7.0]	7.0 [4.5–8.5]	0.592	0.00 [−0.04–0.21]
Shooting (score)	4.0 [3.5–5.0]	2.0 [1.0–3.0]	3.0 [2.5–6.0]	0.336	0.05 [−0.04–0.30]
Pass Shooting (score)	3.0 [2.0–5.5]	5.0 [3.5–8.5]	9.0 [4.0–11.5]	0.336	0.05 [−0.03–0.27]
Heading (front) (score)	0.0 [0.0–0.5]	1.0 [0.0–4.0]	6.0 [3.0–8.5]	**0.016**	0.25 [0.07–0.51]
Heading (side) (score)	0.0 [0.0–2.0]	2.0 [1.0–4.0]	5.0 [1.5–9.0]	0.174	0.12 [−0.01–0.36]

**Table 2:** Rotated component loadings from the PCA after oblimin rotation. Only loadings ≥ |0.40| are shown.

Variable	PC1	PC2	PC3
Sprint 10 m	-	0.93	-
Sprint 30 m	-	0.92	-
YYIRT1	-	0.73	-
CMJ	-	0.70	-
Harre circuit test	0.60	0.42	-
Foot Juggling	0.93	-	-
Body Juggling	0.80	-	-
Speed Dribbling	0.68	-	-
Long Passing	0.71	-	-
Short Passing	-	-	0.59
Shooting	0.50	-	0.43
Pass Shooting	-	-	0.76
Heading (front)	-	-	0.53
Heading (side)	-	-	0.85

## Data Availability

The data are not publicly available due to privacy and ethical restrictions and will be available from the corresponding author upon reasonable request.

## Supplementary Materials

The following supporting information can be downloaded at https://www.mdpi.com/article/10.3390/sports14060233/s1, Figure S1: Scree plot based on the Spearman correlation matrix showing eigenvalues for each principal component and the cumulative explained variance. The dashed horizontal line represents the Kaiser criterion (eigenvalue = 1); Table S1: Spearman correlations among performance variables (n = 49). Rho values with confidence intervals and Benjamini-Hochberg adjusted *p*-values are reported; Table S2: Complete rotated loading matrix from the PCA performed using Spearman correlations and oblimin rotation. Bootstrap-estimated mean loadings derived from 1000 resampling iterations are also reported to provide an exploratory assessment of component-loading stability.
