# Supplementary material for: Interrelationships Among Physical Fitness, General Motor Coordination, and Soccer-Specific Technical Skills in Youth Soccer Players
Source: Sports (Basel). 2026 Jun 5;14(6):233. doi: 10.3390/sports14060233 (PMC13307169; doi:10.3390/sports14060233)

Figure S1. Scree plot based on the Spearman correlation matrix showing eigenvalues for each principal component and the cumulative explained variance. The dashed horizontal line represents the Kaiser criterion (eigenvalue = 1).

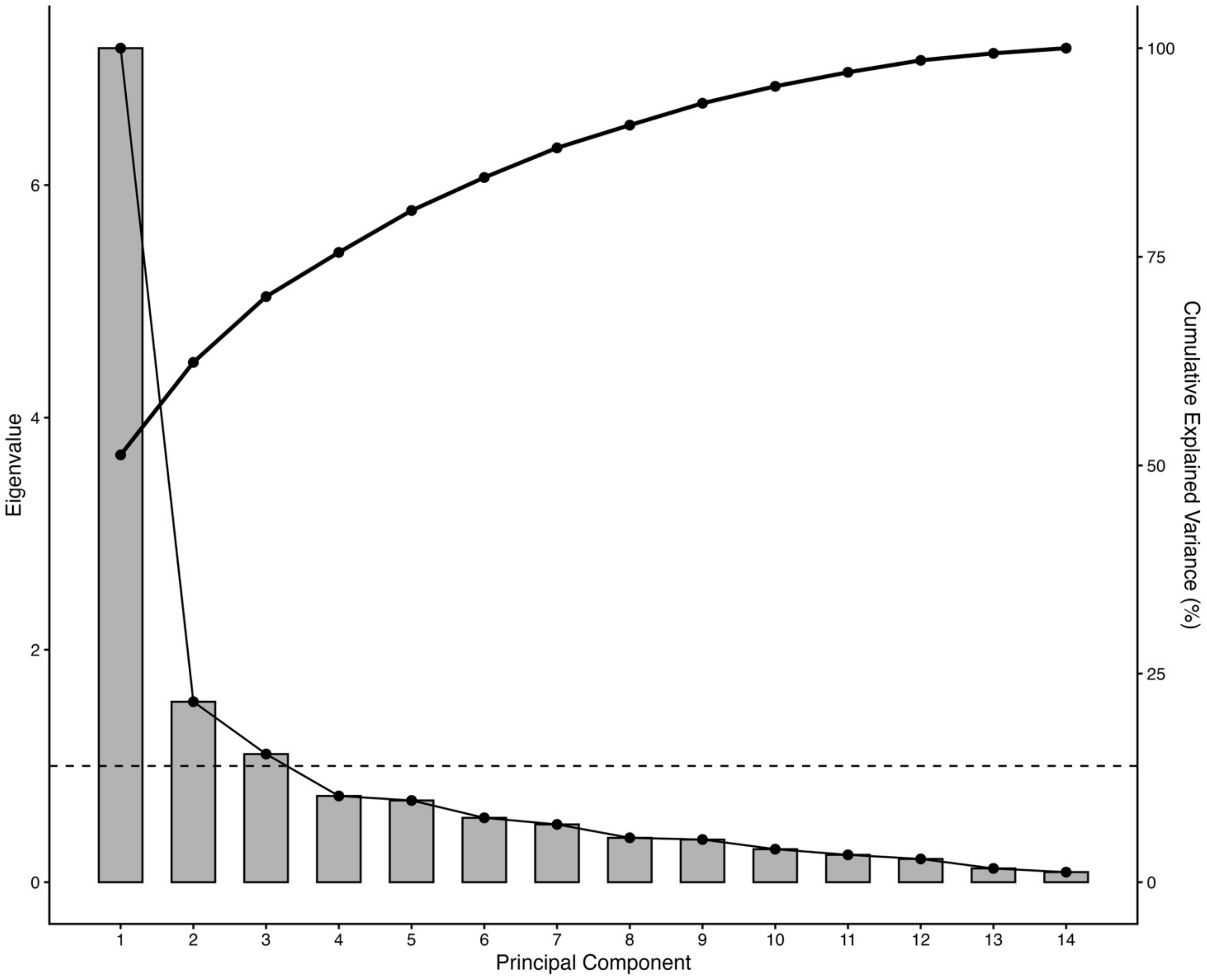

Supplement: Supplementary file 1 [file sports-14-00233-s001.zip › Figure S1.pdf]
