# Supplementary material for: Interrelationships Among Physical Fitness, General Motor Coordination, and Soccer-Specific Technical Skills in Youth Soccer Players
Source: Sports (Basel). 2026 Jun 5;14(6):233. doi: 10.3390/sports14060233 (PMC13307169; doi:10.3390/sports14060233)
Supplement: Supplementary file 1 [file sports-14-00233-s001.zip › Table S1.pdf]

Table S1. Spearman correlations among performance variables (n=49). Rho values with confidence intervals and Benjamini-Hochberg adjusted p-values are reported.

Variable 1	Variable 2	Rho	95% CI	p_adj_BH
10m Sprint	30m Sprint	0.85	0.74-0.91	<0.001
10m Sprint	YYIRT1	0.61	0.39-0.76	<0.001
10m Sprint	CMJ	0.67	0.47-0.80	<0.001
10m Sprint	Harre	0.46	0.20-0.66	0.002
10m Sprint	Foot Juggling	0.26	0.03-0.51	0.069
10m Sprint	Body Juggling	0.44	0.17-0.65	0.003
10m Sprint	Speed Dribbling	0.60	0.38-0.76	<0.001
10m Sprint	Long Passing	0.37	0.09-0.59	0.012
10m Sprint	Short Passing	0.36	0.08-0.59	0.014
10m Sprint	Shooting	0.26	0.03-0.51	0.069
10m Sprint	Pass Shooting	0.23	0.07-0.48	0.120
10m Sprint	Heading (Front)	0.44	0.17-0.65	0.003
10m Sprint	Heading (Side)	0.26	0.03-0.51	0.069
30m Sprint	YYIRT1	0.64	0.43-0.78	<0.001
30m Sprint	CMJ	0.71	0.53-0.83	<0.001
30m Sprint	Harre	0.56	0.32-0.73	<0.001
30m Sprint	Foot Juggling	0.27	0.03-0.52	0.068
30m Sprint	Body Juggling	0.39	0.11-0.61	0.008
30m Sprint	Speed Dribbling	0.60	0.38-0.76	<0.001
30m Sprint	Long Passing	0.41	0.14-0.63	0.005
30m Sprint	Short Passing	0.43	0.16-0.64	0.004
30m Sprint	Shooting	0.38	0.11-0.61	0.009
30m Sprint	Pass Shooting	0.29	0.00-0.54	0.045
30m Sprint	Heading (Front)	0.40	0.13-0.62	0.007
30m Sprint	Heading (Side)	0.31	0.02-0.55	0.036
YYIRT1	CMJ	0.60	0.38-0.76	<0.001
YYIRT1	Harre	0.68	0.49-0.81	<0.001
YYIRT1	Foot Juggling	0.41	0.14-0.63	0.005
YYIRT1	Body Juggling	0.37	0.09-0.6	0.011
YYIRT1	Speed Dribbling	0.57	0.34-0.74	<0.001
YYIRT1	Long Passing	0.44	0.18-0.65	0.003
YYIRT1	Short Passing	0.32	0.04-0.56	0.027
YYIRT1	Shooting	0.32	0.04-0.56	0.028
YYIRT1	Pass Shooting	0.41	0.13-0.62	0.006
YYIRT1	Heading (Front)	0.37	0.10-0.60	0.011
YYIRT1	Heading (Side)	0.27	0.02-0.52	0.068
CMJ	Harre	0.54	0.30-0.72	<0.001
CMJ	Foot Juggling	0.40	0.12-0.61	0.007
CMJ	Body Juggling	0.38	0.10-0.60	0.010
CMJ	Speed Dribbling	0.48	0.23-0.68	0.001
CMJ	Long Passing	0.52	0.27-0.70	<0.001
CMJ	Short Passing	0.51	0.26-0.70	<0.001
CMJ	Shooting	0.38	0.11-0.61	0.009
CMJ	Pass Shooting	0.44	0.17-0.65	0.003

CMJ	Heading (Front)	0.53	0.28-0.71	<0.001
CMJ	Heading (Side)	0.42	0.15-0.64	0.004
Harre	Foot Juggling	0.54	0.30-0.72	<0.001
Harre	Body Juggling	0.55	0.31-0.72	<0.001
Harre	Speed Dribbling	0.73	0.56-0.84	<0.001
Harre	Long Passing	0.58	0.35-0.74	<0.001
Harre	Short Passing	0.49	0.24-0.69	0.001
Harre	Shooting	0.42	0.14-0.63	0.005
Harre	Pass Shooting	0.36	0.08-0.59	0.012
Harre	Heading (Front)	0.38	0.10-0.60	0.010
Harre	Heading (Side)	0.31	0.02-0.55	0.034
Foot Juggling	Body Juggling	0.62	0.41-0.77	<0.001
Foot Juggling	Speed Dribbling	0.70	0.51-0.82	<0.001
Foot Juggling	Long Passing	0.66	0.45-0.79	<0.001
Foot Juggling	Short Passing	0.53	0.29-0.71	<0.001
Foot Juggling	Shooting	0.53	0.28-0.71	<0.001
Foot Juggling	Pass Shooting	0.36	0.08-0.59	0.013
Foot Juggling	Heading (Front)	0.37	0.09-0.60	0.011
Foot Juggling	Heading (Side)	0.32	0.04-0.56	0.028
Body Juggling	Speed Dribbling	0.68	0.49-0.81	<0.001
Body Juggling	Long Passing	0.58	0.35-0.75	<0.001
Body Juggling	Short Passing	0.48	0.22-0.67	0.001
Body Juggling	Shooting	0.48	0.22-0.67	0.001
Body Juggling	Pass Shooting	0.38	0.11-0.61	0.009
Body Juggling	Heading (Front)	0.53	0.28-0.71	<0.001
Body Juggling	Heading (Side)	0.27	0.02-0.52	0.066
Speed Dribbling	Long Passing	0.59	0.37-0.75	<0.001
Speed Dribbling	Short Passing	0.62	0.41-0.77	<0.001
Speed Dribbling	Shooting	0.58	0.35-0.74	<0.001
Speed Dribbling	Pass Shooting	0.45	0.19-0.66	0.002
Speed Dribbling	Heading (Front)	0.50	0.25-0.69	0.001
Speed Dribbling	Heading (Side)	0.42	0.16-0.64	0.004
Long Passing	Short Passing	0.57	0.34-0.74	<0.001
Long Passing	Shooting	0.56	0.33-0.73	<0.001
Long Passing	Pass Shooting	0.31	0.02-0.55	0.036
Long Passing	Heading (Front)	0.56	0.33-0.73	<0.001
Long Passing	Heading (Side)	0.39	0.12-0.61	0.008
Short Passing	Shooting	0.53	0.28-0.71	<0.001
Short Passing	Pass Shooting	0.52	0.27-0.70	<0.001
Short Passing	Heading (Front)	0.47	0.21-0.67	0.001
Short Passing	Heading (Side)	0.59	0.36-0.75	<0.001
Shooting	Pass Shooting	0.39	0.11-0.61	0.008
Shooting	Heading (Front)	0.48	0.23-0.68	0.001
Shooting	Heading (Side)	0.49	0.23-0.68	0.001
Pass Shooting	Heading (Front)	0.56	0.32-0.73	<0.001
Pass Shooting	Heading (Side)	0.47	0.21-0.67	0.001
Heading (Front)	Heading (Side)	0.37	0.09-0.60	0.011
