# Supplementary material for: Interrelationships Among Physical Fitness, General Motor Coordination, and Soccer-Specific Technical Skills in Youth Soccer Players
Source: Sports (Basel). 2026 Jun 5;14(6):233. doi: 10.3390/sports14060233 (PMC13307169; doi:10.3390/sports14060233)
Supplement: Supplementary file 1 [file sports-14-00233-s001.zip › Table S2.pdf]

Table S2. Complete rotated loading matrix from the PCA performed using Spearman correlations and *oblimin* rotation. Bootstrap-estimated mean loadings derived from 1000 resampling iterations are also reported to provide an exploratory assessment of component-loading stability.

**Variable**	**PC1**		**PC2**		**PC3**	
	Original loading	*Bootstrap mean*	*Original loading*	*Bootstrap mean*	*Original loading*	*Bootstrap mean*
10m Sprint	-0.02	-0.05	**0.93**	**0.89**	-0.04	0.04
30m Sprint	-0.02	-0.05	**0.92**	**0.88**	0.04	0.02
YYIRT1	0.21	0.09	**0.73**	**0.51**	-0.05	0.25
CMJ	-0.03	0.01	**0.70**	**0.70**	0.33	0.16
Harre	**0.60**	**0.37**	**0.42**	**0.52**	-0.10	-0.06
Foot Juggling	**0.93**	**0.74**	-0.14	-0.01	0.01	0.05
Body Juggling	**0.80**	**0.70**	0.05	0.05	-0.01	0.07
Speed Dribbling	**0.68**	**0.55**	0.29	0.31	0.07	0.11
Long Passing	**0.71**	**0.40**	0.05	0.06	0.15	0.41
Short Passing	0.33	0.30	0.03	0.08	**0.59**	**0.51**
Shooting	**0.50**	**0.44**	-0.09	-0.07	**0.43**	**0.42**
Pass Shooting	0.00	0.06	0.06	-0.01	**0.76**	**0.71**
Heading (Front)	0.19	0.23	0.18	0.04	**0.53**	**0.54**
Heading (Side)	-0.06	0.01	0.00	0.03	**0.85**	**0.69**
